# Use of DAVID algorithms for clustering custom annotated gene lists in a non-model organism, rainbow trout

**DOI:** 10.1186/s13104-018-3154-7

**Published:** 2018-01-23

**Authors:** Hao Ma, Guangtu Gao, Gregory M. Weber

**Affiliations:** National Center for Cool and Cold Water Aquaculture, Kearneysville, WV 25430 USA

**Keywords:** Gene functional classification, *kappa* statistics, Fuzzy heuristic partition, Soft clustering

## Abstract

**Objective:**

The DAVID gene functional classification tool requires adaptations for use in non-model species and there is little available information to guide selection of a *kappa* score. Our objective was to develop an R-script that allows custom gene identifiers and novel annotation information to be incorporated into analyses, then use such data to evaluate the number of differentially expressed genes (DEGs) in a comparison based on *kappa* score selection.

**Results:**

Using an R-script we developed and multiple data sets ranging from 555 to 3340 annotated DEGs from a study in rainbow trout, we found the percentage of DEGs harbored within a module and the number of genes shared among multiple modules decreased with increasing *kappa* score regardless of the number of DEGs in the comparison. The number of genes in enriched modules peaked at a *kappa* score of 0.5 for the comparisons with 3340 and 1313 DEGs and 0.3 for 555 DEGs. The number of genes harbored within enriched modules generally decreased with increasing *kappa* score; however, this was affected by whether the largest modules were significantly enriched. Large non-enriched modules can be reanalyzed using a higher *kappa* score resulting in some of the genes clustering in smaller enriched modules.

**Electronic supplementary material:**

The online version of this article (10.1186/s13104-018-3154-7) contains supplementary material, which is available to authorized users.

## Introduction

Data analysis software program packages designed to conduct cluster analysis of genes derived from sequencing or microarray data is an essential step to identify functional modules and reveal biological insights [1]. Soft clustering that can assign one gene to multiple clusters has been extensively used in gene analysis [2]. Soft clustering can use gene expression values [3] as well as gene ontology (GO) and other gene or protein annotation sources [4, 5]. This approach takes advantage of the assumption that genes with similar annotation profiles have similar functions [5–7]. Challenges exist with the publicly available programs to conduct soft clustering when atypical annotation sources are required. Many programs are web based and the input format and data resources cannot be changed by users [4, 5, 8–12]. This is a particular problem when the annotation resources used in the program are not updated in a timely manner [13]. Many programs were designed for specific model organisms [5, 8, 14] and don’t provide the flexibility to analyze data derived from non-model organisms. Lastly, the selection of parameters and statistical tests are limited for some software [12, 14, 15].

The web based software DAVID has become one of the most frequently cited tools for gene functional analysis [13, 16]. This tool was initially designed for human, mouse, rat, and fruit fly genomes and has been adopted for use in other species [17, 18]; however, it cannot be used for minor species when custom gene identifiers and their novel transcription information need to be incorporated into the analysis [19]. Furthermore, there have been reports of the gene annotation databases at times being outdated [5, 13, 20]. When using the DAVID gene functional classification tool, the selection of the *kappa* score greatly affects how genes are clustered. *Kappa* statistics, which measures inter-rater agreement, has been shown to be a reliable measurement of the functional gene–gene relationships in DAVID’s algorithm when an appropriate *kappa* value is utilized [5]. The optimal *kappa* score for generation of modules with biologically significant relationships is dependent on the nature of the data set. Thus, selecting an appropriate *kappa* score for a specific data set is a critical step in the data analysis.

The present paper describes the implementation of the agglomeration algorithm behind the DAVID functional gene classification tool with use of custom gene identifiers and their novel transcription information for cluster analysis of differentially expressed genes (DEGs) from a study on rainbow trout. We wrote an R-script to allow a standalone version of the functional gene classification program with which one can directly apply the algorithm to any species using the latest updated resources, and without a limit on input gene identifiers. Using this program, we explore the impact of *kappa* statistics on clustering rainbow trout gene expression data for three comparisons with widely different numbers of DEGs.

## Main text

### Methods

The rainbow trout used in the study were about 2-years-old and from stocks maintained at the USDA National Center for Cool and Cold Water Aquaculture (NCCCWA, Kearneysville, WV). Fish were reared indoors under artificial ambient photoperiod, in continuous-flow treated spring water, at 13 ± 1 °C. Follicle enclosed oocytes from rainbow trout competent to undergo the resumption of meiosis in response to the maturation inducing hormone (MIH), 17α-20β-dihydroxy-4 pregnen-3-one, were incubated in vitro for 24 h with or without either MIH or salmon pituitary homogenate (SPH). Sample total RNA was isolated from follicles freshly collected from the fish (Fresh), follicles cultured without hormone treatment (Control), and follicles cultured with MIH or SPH treatment using Trizol reagent (Invitrogen, Carlsbad, CA) followed by lithium chloride precipitation. Libraries from twelve RNA samples, three replications per treatment, were constructed with TruSeq mRNA Preparation for GAIIx/HiSeq, and then sequenced in 6 lanes using the Illumina HiSeq 2000 platform. Bowtie2 was used with default settings to align raw sequencing reads to a rainbow trout transcriptome database [21] supplemented with an additional 72 gene sequences of interest selected from Gene Bank [22]. At the false discovery rate (FDR) < 0.05, both DESeq2 [23] and edgeR [24] identified 4239 DEGs for control vs MIH treatment (Control_MIH), 1691 DEGs for control vs freshly excised tissue (Control_Fresh), and 691 DEGs for control vs SPH treatment (Control_SPH) comparisons. Those DEGs were analyzed by Blast2GO software [25, 26], and 3340, 1313, and 555 DEGs were annotated for the three comparisons accordingly.

**Fig. 1:**
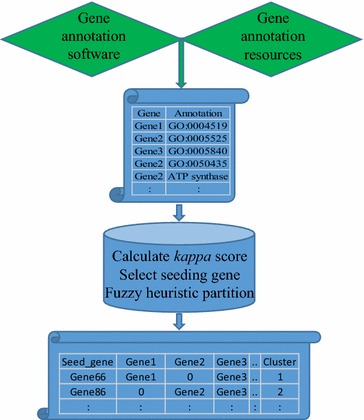
Flowchart of gene functional classification with DAVID’s algorithms in non-model organisms. Steps executed through R-script are in blue

The DEGs with mapped GO terms were input into an R-script with the DAVID gene functional classification algorithms [5] for grouping the genes into functionally related clusters. The script first calculates the *kappa* score to measure the degree of annotated gene pair co-occurrence, then searches for seeding genes, and then conducts functional clustering (Fig. 1). The R-script was tested with DAVID’s sample data and is available in Additional file 1: R_script for clustering.

Module enrichment scores were generated by calculating the geometric mean of the P-values which were derived from hypergeometric test on the input gene sets followed by negative log transformation of the geometric mean. The described rainbow trout transcriptome served as the reference genes used for the hypergeometric test. Pearson’s correlation coefficients were calculated using the R program.

### Results

#### Number of modules and harbored genes

**Fig. 2:**
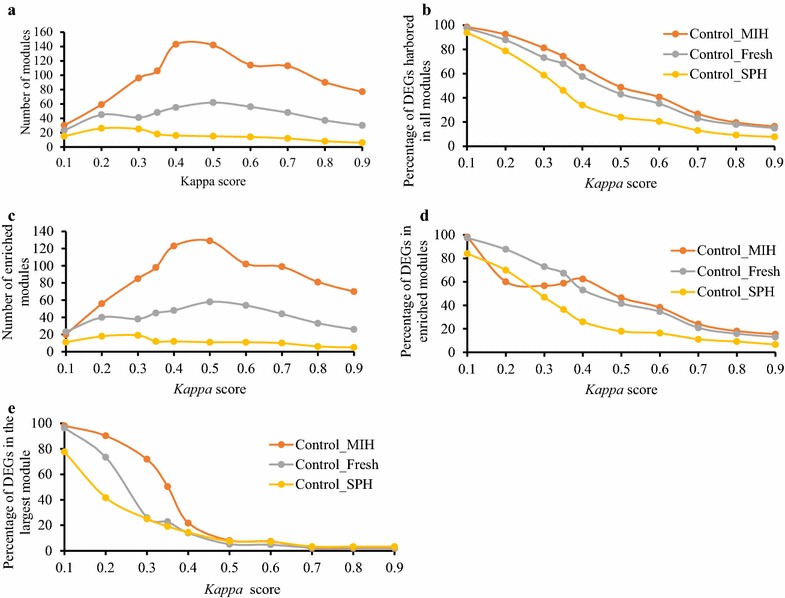
Changes in the number of modules (**a**), percentage of DEGs harbored in all modules (**b**), the number of gene set enriched modules (**c**), percentage of genes harbored in significantly enriched modules (**d**), and the percentage of differentially expressed genes harbored within the largest module (**e**) with *kappa* score

Cluster numbers were dynamically changed under different *kappa* scores for all comparisons (Fig. 2a). The number of modules for Control_MIH and Control_SPH increased as increasing *kappa* scores subdivided modules, but then decreased as fewer gene pairs met the increasing stringency. As would be expected, the peak number of modules was observed at a greater *kappa* score for Control_MIH with 3340 DEGs, than Control_SPH with 555 DEGs. The percentage of DEGs harbored in all modules decreased with increased *kappa* score and decreased at a greater rate as the number of DEGs in the comparison decreased (Fig. 2b).

#### Genes shared by different modules

As an individual gene may be involved in multiple biological functions, it is reasonable that these multi-function genes are shared by multiple modules with each module composed of genes associated with a disparate function. In our data sets, the number of genes harbored in multiple modules decreased as *kappa* score increased (Table 1). The percent of genes clustered in multiple modules also decreased with increasing *kappa* score as the number of DEGs in the comparison decreased. Less than 10% of the genes clustered to multiple modules when the *kappa* score exceeded 0.5 for Control_MIH, 0.4 for Control_Fresh, and 0.3 for Control_SPH.

**Table 1:** Changes in the number of genes shared among different numbers of modules with *kappa* score

Comparison	Number of modules	Number of genes shared among modules									
		K = 0.1	K = 0.2	K = 0.3	K = 0.35	K = 0.4	K = 0.5	K = 0.6	K = 0.7	K = 0.8	K = 0.9
Control_MIH	1	861	1145	985	1128	1539	1443	1229	883	650	548
	2	1511	1384	1167	847	486	158	110	2	0	0
	3	746	436	415	343	110	23	17	4	0	0
	4	146	105	117	131	23	2	0	0	0	0
	5	24	20	16	27	16	1	0	0	0	0
	6	1	0	4	5	2	0	0	0	0	0
	7	0	0	9	2	0	0	0	0	0	0
Control_Fresh	1	431	391	585	704	657	536	444	302	236	197
	2	653	439	264	147	83	25	15	0	0	0
	3	172	235	91	34	11	4	4	0	0	0
	4	22	70	17	9	6	0	0	0	0	0
	5	1	14	3	1	0	0	0	0	0	0
	6	0	5	1	0	0	0	0	0	0	0
Control_SPH	1	201	282	281	236	184	132	113	71	51	43
	2	224	101	39	20	5	1	1	1	0	0
	3	72	44	6	0	0	0	0	0	0	0
	4	19	9	0	0	0	0	0	0	0	0
	5	5	1	0	0	0	0	0	0	0	0

#### Number of enriched modules

Another important factor in gene functional classification is the enrichment score of modules, which helps to identify the most biologically relevant gene clusters. Nevertheless, some modules with an enrichment score of less than 1.3 (P < 0.05) could be potentially interesting [15]. The number of enriched modules combining all three GO categories peaked at a *kappa* score of about 0.5 for Control_MIH and Control_Fresh, but peaked earlier at 0.3 for Control_SPH which had the least number of DEGs (Fig. 2c). However, the total number of genes harbored among enriched modules generally decreased drastically with increasing *kappa* score (Fig. 2d). The Pearson’s correlations between the *kappa* scores and number of genes harbored in the enriched modules were − 0.947, − 0.983, and − 0.914 for Control_MIH, Control_Fresh, and Control_SPH respectively, and were highly significant (P < 0.001).

#### Cluster size

Implementing the fuzzy heuristic multiple-linkage partition in DAVID often resulted in one extremely large cluster of DEGs when low *kappa* scores were applied (Additional file 2: Table S1, module 1). The percentages of the DEGs in the largest module decreased dramatically with increased *kappa* score, and decreased more rapidly as the number of DEGs in the comparisons decreased (Fig. 2e). When using the data for all three GO categories, the enrichment scores of the largest modules were significant for all comparisons for all *kappa* scores except *kappa* scores below 0.4 for Control_MIH (Additional file 2: Table S2). Enrichment scores for the largest module increased consistently with *kappa* score for Control_MIH, but peaked at mid *kappa* scores for Control_Fresh and Control_SPH. This pattern held for Control_MIH when looking at data for the GO categories individually, but the patterns were less consistent among GO categories for Control_Fresh and Control_SPH (Additional file 2: Table S3).

When the number of DEGs harbored in the largest module is high and the DEGs in the module is not significantly enriched, such as in the combined GO category data for Control_MIH *kappa* scores 0.1–0.35, with 3279–1684 genes, respectively (Additional file 2: Table S2); the total number of genes harbored among enriched modules can be reduced relative to higher *kappa* scores (Fig. 2d). Thus, the ability to identify interactions of those genes which are not found in enriched modules with our other DEGs, is reduced in the analysis. One strategy to generate significantly enriched gene clusters for genes within these large modules is to break down the large module into smaller sub-modules by using a higher *kappa* score. We tested this at *kappa* score of 0.35 for Control_MIH. Using *kappa* score 0.35 for the complete data set (3340 DEGs), the largest module contains 1684 DEGs (see Additional file 2: Table S2), among which 449 DEGs are not found in other modules that were significantly enriched. However, at *kappa* score 0.6, this large module yielded 74 sub-modules of which 18 contained a total of 250 of the genes that were not previously incorporated into any enriched module. Seventeen of these 18 sub-modules were significantly enriched and contained 246 of the DEGs not incorporated into enriched modules in the initial analysis using *kappa* 0.35.

### Discussion

Our R-script provides a flexible way to conduct gene functional cluster analysis for model and non-model organisms with DAVID’s algorithms [5] (Fig. 1). When using this program, there is no restriction to input annotated gene identifiers. In addition, users can prepare a flat matrix by using any software or laboratory experiment to get desired information from any gene resource.

Clustering of data sets into modules in which the genes have a functional relationship is highly dependent on the *kappa* score used in the analysis [5, 15]. In general, as the *kappa* score is increased the number of genes in the largest modules decreases. As the number of genes in a module decreases, the shared function of those genes becomes more specific; however, modules with few genes can only provide insight into the interactions of those limited numbers of genes. Thus, an investigator must choose a stringency or *kappa* score that is appropriate for their data set. Some papers report a *kappa* score of 0.35 [17, 27] as suggested in the DAVID program, but many papers either don’t mention the *kappa* score [18, 28–35] or report using scores above 0.35; e.g. 0.45 [36], 0.5 [37–39], 0.80 [40], or even 0.85 [41]. Few publications provide information on how or why *kappa* scores were selected.

In our RNA-seq data analysis, the number of modules in the comparison with the greatest number of DEGs (Control_MIH) was observed at a much higher *kappa* value than for the comparison with the least number of DEGs (Control_SPH) (Fig. 2a). As mentioned, the number of modules increases as increasing *kappa* scores subdivides modules with large gene sets, but then the number of modules decreases as more gene pairs fail to meet the increasing stringency. As expected, the more DEGs in the comparisons the greater the likelihood of more modules with large gene sets at low *kappa* scores (Additional file 2: Table S1). Regardless of the number of DEGs in a comparison, the percentage of genes harbored in all modules decreased as *kappa* score increased presumably as more gene pairs failed to meet stringency (Fig. 2b). Similar patterns were observed in terms of how *kappa* score affected the number of enriched modules and the percentage of genes harbored within enriched modules (Fig. 2c, d).

### Limitations

Although we used multiple data sets ranging from 555 to 3340 annotated genes in rainbow trout to test the impact of *kappa score* on functional gene cluster analysis, the results only serve as a guide for the impacts of changes in data set size. Actual results will likely be impacted by differences in the size and diversity of the transcriptome among tissues, reference genome annotation, and species.

## Additional files

**Additional file 1.** R_script for clustering.

**Additional file 2: Table S1.** Changes in the number of genes harbored in each module with *kappa* score. **Table S2.** The enrichment score for the largest module estimated by using three GO categories. **Table S3.** Changes in the enrichment score for the largest module with *kappa* score, estimated for each GO category.

## Abbreviations

DEGs: differentially expressed genes
MIH: maturation inducing hormone
SPH: salmon pituitary homogenate
GO: gene ontology
